# The comparison of digestibility of treated sugarcane tops silage by bacteria or whole microorganisms of Holstein cow and buffalo rumen

**Published:** 2016-09-15

**Authors:** Afrooz Sharifi, Morteza Chaji, Tahereh Mohammadabadi

**Affiliations:** 1 *Graduate Student of Animal Nutrition, Ramin Agriculture and Natural Resources University of Khouzestan, Ahvaz, Iran; *; 2 *Department of Animal Science, Ramin Agriculture and Natural Resources University of Khouzestan, Ahvaz, Iran.*

**Keywords:** Bacteria, Buffalo, Molasses, Sulfuric acid, Urea

## Abstract

The aim of this study was to evaluate the effects of adding sulfuric acid to sugarcane tops silage on rumen bacteria and whole rumen microorganisms (WRM) and compare the digestibility of sugarcane tops treated with different amount of urea, molasses and sulfuric acid between Holstein cow and Khouzestan buffalo. Regardless of the type of the treatment, potential of gas production (B) by cow WRM (130.670 mL) was more than buffalo (104.060 mL) (*p *< 0.05), but the rate of gas production (C) by buffalo WRM was greater than cow (0.021 and 0.014 mL per hr, respectively) (*p *< 0.05). The C in treatment containing only 2.40% sulfuric acid (0.033 mL per hr) was significantly highest (*p *< 0.05). Regardless of the type of the treatment, the B by cow rumen bacteria (75.040 mL) was more than buffalo (67.150 mL), (*p *< 0.05), while the C by rumen bacteria of buffalo (0.030 mL per hr) was more than cow (0.017 mL per hr), (*p *< 0.05). Regardless of the type of the animal, the B coefficient of rumen bacteria in treatment only containing 2.40% sulfuric acid was higher than control (*p *< 0.05). Therefore, the addition of sulfuric acid not only had no negative effect on microorganisms particularly bacteria, but also probably due to present of sulfur in acid, had positive effect on nutrients digestibility, and growth of microorganisms. The digestibility of sugarcane tops silage treated by cow rumen bacteria and whole microorganisms was higher than buffalo.

## Introduction

Cultivation history of sugarcane in southern Iran is very long, especially in Khouzestan province. In Iran, quantity of sugarcane production is over 6,000,000 tons.^1^ Therefore, extraction of sugar from sugarcane produces large quantities of by-products which could be valuable feedstuff for ruminants feeding in dry seasons. Sugarcane tops (SCT) are major by-products of the sugarcane industry which are often left in the field after cane harvest.^2^ Approximately 1.40 million tons of SCT are produced annually in the Khouzestan province. However, the low digestibility and high lignin are considered as main reasons for unsatisfactory performance of animals fed with this roughage.^3^ Considering limited harvest season, high levels of SCT production and low nutritional value of SCT, using silo to preserve (and use in other seasons) and enrich SCT with some additives could be useful.

Generally, silage additives are edible materials including urea, molasses, bacteria inoculants and acids. Molasses as a cheap carbohydrate source for lactic acid bacteria provides necessary sugar and carbohydrate for fermentation process. Many experiments have proved that molasses increases lactic acid fermentation and reduces silage pH.^4^ Digestibility of SCT crude protein is low,^5^ therefore, using a suitable source of nitrogen, which improves sugarcane nutritional value, is useful. These additives (e.g. urea) are useful when they can be easily fermented by microorganisms.^6^ On the other hand, due to slow reduction in pH, it was reported that using urea lonely, is unable to stop proteolysis, entirely.^7^ Although urea + molasses,^4^^,^^5^ or molasses alone,^4^ can improve digestion and storage duration of SCT,^6^ but these additives are not suitable for long term storage of silage.^4^^,^^6^ Previously, formic acid and sulfuric acid have been used to decrease the pH of silage.^8^ It was found that rapid acidification of silage is important, and application of acids leads to rapid decrease of pH to below 4 and prevents proteolysis activity.^8^ However, there is conflicting information about capacity of sulfuric acid to lower the pH and probable negative effect of sulfur on activity of rumen bacteria.^9^^,^^10^ It has been reported that 0.32% sulfur increases the population of the rumen microorganisms.^9^ But in another study, when sulfur (as sodium sulfate) content of diet was 0.30%, the growth of rumen microbes restrained, and synthesis of microbial protein decreased.^10^

The reported populations of microorganism in the rumen of cattle and buffalos have been different.^11^^,^^12^ Many factors such as physiological situation, animal age, feeding behavior, level of production, animal health, the nature and relationships between different microbial populations and also external factors such as diet composition, nature of feed, feed frequency, dietary changes, change of seasons and geographical factors can affect the ratio and density of different groups of rumen microorganisms.^13^

Therefore, the aim of the present study was to assess the digestibility and feeding value of acid sulfuric and molasses + urea treated SCT silage by gas test running with different inoculums from Holstein cow and buffalo.

## Materials and Methods

This experiment was carried out at Ramin Agriculture and Natural Resources University of Khouzestan, Ahvaz, Iran. Sugarcane tops for ensiling were prepared from Amir Kabir Agro-Industry (Ahvaz, Iran). Sugarcane tops were chopped (3 to 5 cm) and ensiled into plastic bags in triplicate (4 kg). Before ensiling, SCT were mixed with respective treatments well and transferred to bags. Then, they packed massively until no air remains inside the bags. After 120 days, the silos were opened. Experimental treatments were: 1) SCT ensiled without additive (control), 2) SCT ensiled with urea (1.00%) + molasses (3.00%), 3) SCT ensiled with sulfuric acid 2.40% and 4) SCT ensiled with urea (1.00%) + molasses (3.00%) and sulfuric acid (2.40%).

Gas production (GP) experiments were run three times, each run as a repeat. Rumen fluid was collected from two fistulated cattle (weighted 430 ± 12 kg) and buffalo steer (weighted 420 ± 14 kg) before morning feeding. They were fed twice per day with maintenance diet (including alfalfa hay, wheat straw, sugarcane pith, soybean meal, barley, corn, urea, minerals and vitamin materials). Collected rumen liquid was strained through four layers of cheesecloth and mixed with an appropriate volume of artificial saliva.

Gas production of experimental treatments by whole rumen microorganisms (WRM) was measured according Menk and Stingenss method in 100 mL glass syringes containing 300 mg of ground sample, 20 mL of artificial saliva and 10 mL of rumen liquid. ^14^ The same method was used to determine GP of rumen bacteria, but for isolation of rumen bacteria, after collection and straining of liquor, rumen fluid was centrifuged (1000 rpm for 10 min) for removing protozoa. Then, bacteria were isolatedfrom non-protozoa strained rumen fluid using antifungal agents benomyle (500 mg L^-1^) and metalaxyle (10 mg L^-1^).^15^ Gas production was measured after 2, 4, 6, 8, 12, 16, 24, 36, 48, 72 and 96 hr. For measurement of cell wall degradability, the syringes contents were carefully filtered (No. 42; Whatman, Pittsburgh, USA) and then, the residues were washed with distilled water into a tube, separately. Then, the residues were dried at 105 ˚C for 12 hr and used to calculate the degradation of samples. Cumulative GP data were fitted to the exponential equation:

 Y=b (1-e^-ct^)

where, b is GP from fermentable fraction (mL), c is GP rate constant (mL per hr), t is the incubation time (hr) and Y is the gas produced at time t.^16^

To estimate partitioning factor (PF), which expresses as ratio of truly degraded organic matter to gas produced in incubation periods,^17^ at the end of incubation period, the content of each syringe was transferred into Erlenmeyer flask (Schott, Mainz, Germany), mixed with 20 mL neutral detergent fiber solution, boiled for 1 hr and then, filtered, oven-dried at 60 ˚C for 48 hr (Memmert, Schwabach, Germany) and ashed in furnace at 550 ˚C for 3 hr (Exciton, Tehran, Iran). Partitioning factor, microbial biomass and actually degradable organic matter were calculated by Makkar method.^18^

Data were analyzed by split plot design, main plot was animal species (cattle or buffalo) and subplot included microorganism type (WRM or bacteria). Using general linear model procedures of SAS (Version 9.1; SAS Institute, Cary, USA), Duncan’s multiple range tests were used to compare the means.

## Results

Gas production by WRM. In buffalo, the highest potential of GP was for SCT ensiled with 2.40 % sulfuric acid (134.83 mL) and in cattle was for control treatment (146.56 mL), (p < 0.05). The highest rate of GP in buffalo was for SCT ensiled with sulfuric acid (0.045 mL per hr) and for cattle was in urea + molasses + sulfuric acid (0.021 mL per hr) treatment (p < 0.05), (Table 1). Regardless of the type of the treatment, potential of GP by WRM of cattle was significantly (p < 0.05) higher than buffalo (130.670 and 104.060 mL, respectively), but GP rate was significantly (p < 0.05) higher in buffalo (0.021 and 0.014 mL per hr, respectively), (Table 1). Regardless of the type of the animal, the effect of different processing methods on potential of GP of SCT silage was not significant. But, numerically the highest potential and rate of GP (123.940 mL, 0.033 mL per hr, respectively) were in the treatment containing 2.40% sulfuric acid (Table 1).

**Table 1:** Gas production parameters of treated sugarcane tops silage by whole rumen microorganisms of cattle and buffalo

**Treatment**	**Animal**	**Urea + Molasses (%)**	**Sulfuric acid (%)**	**Potential of gas production (mL)**	**Gas production rate (mL hr** ^-1^ **)**
**1**	Buffalo	0.00	0.00	81.000^b^	0.004^d^
**2**		1.00 + 3.00	0.00	89.600^b^	0.004^d^
**3**		0.00	2.40	134.830^a^	0.045^a^
**4**		1.00 + 3.00	2.40	110.130^ab^	0.031^b^
**1**	Cattle	0.00	0.00	146.560^a^	0.006^d^
**2**		1.00 + 3.00	0.00	145.180^a^	0.009^d^
**3**		0.00	2.40	113.050^ab^	0.020^c^
**4**		1.00 + 3.00	2.40	115.870^ab^	0.021^c^
**SEM**				10.980	0.002
***p*** **-value**				0.0045	0.0001
***Regardless of the type of the treatment***					
**Buffalo**				104.060^b^	0.021^a^
**Cattle**				130.170^a^	0.014^b^
**SEM**				5.490	0.0010
***p*** **-value**				0.0040	0.0001
***Regardless of the type of the animal***					
**1**		0.00	0.00	114.120	0.005^c^
**2**		1.00 + 3.00	0.00	117.390	0.007^c^
**3**		0.00	2.40	123.940	0.033^a^
**4**		1.00 + 3.00	2.40	113.000	0.026^b^
**SEM**				7.760	0.0014
***p*** **-value**				0.7540	0.0001

**SEM:** Standard error of the means; Different superscripts within each column indicate significant differences (*p *< 0.05).

Gas production by rumen bacteria. The effect of different treatments on GP of SCT silage by rumen bacteria was significant (Table 2). In buffalo, the highest potential of GP was in the treatment containing 2.40% sulfuric acid (p < 0.05). In buffalo, GP rate of SCT ensiled with 2.40% sulfuric acid (0.068 mL per hr), and in cattle, GP rate of SCT ensiled with urea + molasses + sulfuric acid (0.025 mL per hr) was the highest amount (p < 0.05). Regardless of the type of the treatment, potential of GP by rumen bacteria of cattle was significantly higher than buffalo (75.040 and 167.150 mL, respectively). Reversely, GP rate in buffalo was higher (0.030 and 0.017 mL per hr, respectively; p < 0.05), (Table 2). Regardless of the type of the animal, the potential of GP by the rumen bacteria for all treatments was higher than control (p < 0.05). The rate of GP in treatments containing 2.40% sulfuric acid (0.046 mL per hr) and urea + molasses + sulfuric acid (0.035 mL per hr) was significantly more than control (0.006 mL per hr; (p < 0.05), (Table 2).

**Table 2:** Gas production parameters of treated sugarcane tops silage by rumen bacteria of cattle and buffalo.

**Treatment**	**Animal**	**Urea + Molasses (%)**	**Sulfuric acid (%)**	**Potential of gas production (mL)**	**Gas production rate (mL hr** ^-1^ **)**
**1**	Buffalo	0.00	0.00	47.740^b^	0.005^ef^
**2**		1.00 + 3.00	0.00	71.200^a^	0.002^f^
**3**		0.00	2.40	78.010^a^	0.068^a^
**4**		1.00 + 3.00	2.40	71.640^a^	0.045^b^
**1**	Cattle	0.00	0.00	72.940^a^	0.008^de^
**2**		1.00 + 3.00	0.00	73.290^a^	0.012^d^
**3**		0.00	2.40	77.710^a^	0.023^c^
**4**		1.00 + 3.00	2.40	76.230^a^	0.025^c^
**SEM**				4.600	0.001
***p*** **-value**				0.0070	0.0001
***Regardless of the type of the treatment***					
**Buffalo**				67.150^b^	0.030^a^
**Cattle**				75.040^a^	0.017^b^
**SEM**				2.340	0.000
***p*** **-value**				0.030	0.000
***Regardless of the type of the animal***					
**1**		0.00	0.00	60.340^b^	0.006^c^
**2**		1.00 + 3.00	0.00	72.240^a^	0.007^c^
**3**		0.00	2.40	77.860^a^	0.046^a^
**4**		1.00 + 3.00	2.40	73.940^a^	0.035^b^
**SEM**				3.320	0.0010
***p*** **-value**				0.0109	0.0001

**SEM:** Standard error of the means; Different superscripts within each column indicate significant differences (*p *< 0.05).

Gas production parameters by WRM. The results showed that the highest microbial biomass was in diet containing urea + molasses + sulfuric acid (p < 0.05) for both buffalo (50.30 mg) and cattle (62.75 mg). In buffalo, the highest PF and microbial‌ biomass efficiency were in control (4.30 mg mL^-1^, 49.00%, respectively). In cattle, the highest PF and microbial‌ biomass efficiency were in the treatment containing sulfuric acid (3.48 mg mL^-1^ and 37.00%, respectively), (p < 0.05). The highest true organic matter disappearance (TOMD) was in the treatment containing sulfuric acid (171.50 mg) in buffalo, and in cattle, the highest TOMD was in urea + molasses + sulfuric acid treatment (178.25 mg; p < 0.05), (Table 3). Regardless of the type of the treatment, the PF  (p < 0.05) in buffalo (3.46 mg mL^-1^) was higher than cattle (3.21 mg mL^-1^). However, microbial biomass and TOMD of cattle were higher (p < 0.05) than buffalo (Table 3). Regardless of the type of the animal, TOMD and microbial biomass in SCT silage treated with urea + molasses + sulfuric acid (173.12 mg and 56.52 mg, respectively) were higher than other treatments (p < 0.05). The highest PF and microbial biomass efficiency were in control (3.67 mg mL^-1^ and 38.21%, respectively), (p < 0.05) (Table 3).

**Table 3:** Gas production parameters of treated sugarcane tops silage by whole rumen microorganisms of cattle and buffalo

**Treatment**	**Animal**	**Urea +** **Molasses (%)**	**Sulfuric acid (%)**	**PF** * **(mg mL** ^-1^ **)**	**Microbial biomass (mg)**	**Efficiency microbial biomass (%)**	**True organic matter disappearance (mg)**	**Cell wall degradation**
**1**	Buffalo	0.00	0.00	4.30^a^	42.00^bc^	49.00^a^	73.50^d^	54.83
**2**		1.00 + 3.00	0.00	3.83^b^	28.50^dc^	42.00^b^	67.00^c^	64.00
**3**		0.00	2.40	2.58^f^	25.20^d^	15.00^f^	171.50^a^	65.83
**4**		1.00 + 3.00	2.40	3.14^de^	50.30^ab^	30.00^de^	168.00^a^	69.27
**1**	Cattle	0.00	0.00	3.04^e^	29.85^dc^	27.00^e^	107.90^b^	55.07
**2**		1.00 + 3.00	0.00	2.92^e^	24.40^d^	24.00^e^	99.20^b^	61.63
**3**		0.00	2.40	3.48^c^	61.30^a^	37.00^bc^	166.90^a^	67.50
**4**		1.00 + 3.00	2.40	3.39^de^	62.75^a^	35.00^dc^	178.25^a^	71.03
**SEM**				0.0866	4.06	0.0192	4.05	-
***p*** **-value**				0.0001	0.0001	0.0001	0.0001	-
***Regardless of the type of the treatment***								
**Buffalo**				3.46^a^	36.50^b^	33.97	120.00^b^	63.48
**Cattle**				3.21^b^	44.57^a^	30.97	138.07^a^	63.81
**SEM**				0.0433	2.0300	0.0093	3.1300	-
***p*** **-value**				0.0001	0.0001	0.0001	0.0001	-
***Regardless of the type of the animal***								
**1**		0.00	0.00	3.67^a^	35.92^c^	38.21^a^	90.72^b^	54.95
**2**		1.00 + 3.00	0.00	3.37^b^	26.45^b^	33.52^b^	83.10^b^	62.81
**3**		0.00	2.40	3.03^c^	43.25^b^	25.63^b^	169.20^a^	66.66
**4**		1.00 + 3.00	2.40	3.27^b^	56.52^a^	32.52^a^	173.12^a^	70.15
**SEM**				0.0612	2.8700	0.0136	4.4300	-
***p*** **-value**				0.0001	0.0001	0.0001	0.0001	-

**SEM:** Standard error of the means.

Different superscripts within each column indicate significant differences (*p *< 0.05).

Due to lack of repetition, statistical analysis was not done in terms of cell wall degradation.

* **PF:** partitioning factor;

Gas production parameters by rumen bacteria. The highest microbial biomass for buffalo was in SCT ensiled with sulfuric acid (69.10 mg) and in cattle, it was for urea + molasses + sulfuric acid (75.25 mg) treatment. The highest PF and microbial biomass efficiency for buffalo were in control (9.75 mg mL^-1^ and 77.42%, respectively) and for cattle, the highest PF and microbial biomass efficiency were in urea + molasses + sulfuric acid treatment (4.37 mg mL^-1^ and 49.63%, respectively) (p < 0.05). In buffalo, TOMD of acid treatment (145.77 mg) and in cattle, TOMD of urea + molasses + sulfuric acid treatment (151.51 mg) had the highest values (p < 0.05), (Table 4). Regardless of the type of the treatment, PF, microbial biomass efficiency and microbial biomass by buffalo were more than cattle (p < 0.05). The TOMD in cattle was higher than buffalo (p < 0.05) (Table 4). Regardless of the type of the animal, the TOMD and microbial biomass of SCT silage treated with urea + molasses + sulfuric acid (p < 0.05) and PF and microbial biomass efficiency in control (6.88 mg mL^-1^ and 61.30%, respectively), were the highest (p < 0.05) (Table 4).

**Table 4:** Gas production parameters of treated sugarcane tops silage by rumen bacteria of cattle and buffalo

**Treatment**	**Animal**	**Urea +** **Molasses (%)**	**Sulfuric acid (%)**	**PF** * **(mg mL** ^-1^ **)**	**Microbial biomass (mg)**	**Efficiency microbial biomass (%)**	**True organic matter disappearance (mg)**	**Cell wall degradation**
**1**	Buffalo	0.00	0.00	9.75^a^	56.60^c^	77.42^a^	62.47^c^	46.53
**2**		1.00 + 3.00	0.00	6.57^b^	37.89^d^	66.53^b^	56.95^c^	50.50
**3**		0.00	2.40	3.78^d^	60.89^bc^	41.73^d^	145.77^a^	55.88
**4**		1.00 + 3.00	2.40	4.26^c^	69.10^ab^	48.34^c^	142.80^a^	58.80
**1**	Cattle	0.00	0.00	4.02^cd^	41.52^d^	45.20^cd^	91.76^b^	46.73
**2**		1.00 + 3.00	0.00	3.08^e^	24.18^e^	28.60^e^	84.32^b^	52.31
**3**		0.00	2.40	4.25^c^	68.52^ab^	48.22^c^	141.86^a^	57.30
**4**		1.00 + 3.00	2.40	4.37^c^	75.25^a^	49.63^c^	151.51^a^	60.30
**SEM**				0.1143	3.4500	0.0154	5.3200	-
***p*** **-value**				0.0001	0.0001	0.0001	0.0001	-
***Regardless of the type of the treatment***								
**Buffalo**				6.09^a^	56.12	58.50^a^	102.00^b^	52.92
**Cattle**				3.93^b^	52.37	42.91^b^	117.36^a^	54.16
**SEM**				0.0571	1.1300	0.0077	2.6600	-
***p*** **-value**				0.0001	0.1636	0.0001	0.0001	-
***Regardless of the type of the animal***								
**1**		0.00	0.00	6.88^a^	49.06^b^	61.31^a^	77.12^b^	46.63
**2**		1.00 + 3.00	0.00	4.83^b^	31.03^c^	47.57^bc^	70.63^b^	51.40
**3**		0.00	2.40	4.02^d^	64.71^a^	44.97^c^	143.82^a^	56.59
**4**		1.00 + 3.00	2.40	4.32^c^	72.17^a^	48.10^b^	147.16^a^	59.55
**SEM**				0.0808	2.4400	0.0109	3.7600	-
***p*** **-value**				0.0001	0.0001	0.0001	0.0001	-

**SEM:** Standard error of the means.

Different superscripts within each column indicate significant differences (*p *< 0.05).

Due to lack of repetition, statistical analysis was not done in terms of cell wall degradation.

* **PF:** partitioning factor;

## Discussion

Treatments containing sulfuric acid or urea + molasses + sulfuric acid had the highest rate of GP, probably due to presence of rapidly fermentable carbohydrates in molasses, increased ammonia gas production by adding urea,^19^ and also neutral detergent fiber (NDF) reduction through degradation of hemicellulose bounds by sulfuric acid.^20^ Our results were in accordance with previous report suggesting that silages treated with hydro chloric acid and urea were more degradable than untreated ones.^21^ It has also been shown that hydrochloric acid increases rapid degradable fraction of alfalfa silage. This finding has been contributed to the inhibitory effect of acid on deleterious aerobic fermentation.^21^ These results were in agreement with the results of Khosropour,^22^ who showed that adding 2.40% sulfuric acid to SCT silage, increases GP rate. Generally, adding urea + molasses + sulfuric acid or sulfuric acid individually, improved nutritional value and utilization of SCT silage by WRM.

Regardless of the type of the treatment (Table 1), potential of GP by WRM of cattle was significantly higher than buffalo, but GP rate was higher in buffalo. The lower GP rate means little GP during the early hours of incubation in cattle, probably due to the fact that fungi have greater role in fiber digestion than other micro-organisms and their colonization is dilatory and needs more time for digestion of feeds and consequent GP.^23^ Results of the present experiment are in agreement with, Shakarami,^12^ who described that GP rate from wheat straw by WRM of buffalo is higher than cattle. Our results were in contrast to findings of Rafiee,^11^ who reported that GP rate of wheat straw by the WRM of buffalo is higher than cattle. The higher potential of GP in cattle compared to buffalo, might be related to higher amounts of fungi in rumen of cattle than buffalo, which have important role in fiber digestion. According to Kumar et al.report,^24^ with diet based on rye grass–concentration, rumen fungi in cattle were higher than buffalo. It has also been reported that for diet based on wheat straw, the rumen fungi of Khouzestan buffalo (2.00 × 10^3^ mL) are lesser than Holstein cattle (2.70 × 10^3 ^mL).^12^ Whereas Chanthakhoun et al.and Wanapat reported that anaerobic fungi of buffalo are more than cattle depending on diet and other conditions.^25^^,^^26^ Fungi are about 8.00% of rumen microbial biomass,^27^ and digest more than 70.00% of cellulose,^28^and about 34.00% of plant tissues lignin.^29^ It has been reported that anaerobic fungi increase feed efficiency and nutrient digestibility of crossbred calves.^30^ Other reasons for the differences in digestion and its improvement in cattle may be attributed to ability of cattle for digestion of cell wall components. It was found that in fibrous ration, NDF and acid detergent fiber (ADF) digestibility in buffalo is lower than cattle, which could be due to better utilization of cellulose in cattle than buffalo.^31^ During feeding with diet based on sorghum, Moran et al. observed same results for Short Horn beef cattle compared to buffalo.^32^‌ Adding concentrate to wheat straw based diet, significantly increased digestibility of dry matter (DM), crude protein and NDF in cattle, while in buffalo only increased the digestibility of crude protein.^33^ Therefore, in the present experiment, in addition to higher fungi population, higher GP in cattle than buffalo, might be because of more ability of cattle for nutrients digestion, especially fiber.

Regardless of the type of the animal (Table 1), the highest potential and rate of GP were numerically in the treatment containing 2.40% sulfuric acid. The reason of increased potential and rate of GP by adding sulfuric acid was probably due to the effect of chemical treatments such as acid for degradation of lignocellulosic linkages between structural components and increasing their bioavailability for microorganisms.^34^ Haddi et al. reported that there is significant negative correlation between NDF and ADF of feed and the rate and potential of GP.^35^ The negative effect of cell wall content on GP could be due to reduction of microbial activity via increasing adverse environmental conditions by duration of incubation time. Since SCT contain high amounts of NDF (76.96%) and acid detergent lignin (6.69%),^36^ the chemical treatment loosens and breakdowns the ester linkages resulting in NDF reduction,^37^ and digestibility and GP rate increase. On the other hand, urea + molasses has high disappearance rate, but when silage was opened, probably little amount of them remains. Thereby, in comparison to control, this treatment had no significant effect on GP rate.

Addition of urea + molasses + sulfuric acid numerically decreased potential of GP compared to control (p > 0.05). Probably this effect was due to high amount of sulfur in medium. By contemporary using of molasses and sulfuric acid (as source containing sulfur), high sulfur resulted in reduction of rumen bacteria growth, especially cellulolytic ones and NDF digestibility. Another reason is probably due to high reduction of rumen pH following simultaneous adding of urea + molasses and sulfuric acid. When rumen pH is lower than 6.20, DM and fiber degradation significantly reduces.^38^

The treatments had significant effect on GP (Table 2) of SCT silage by rumen bacteria of cattle and buffalo. Treatment with sulfuric acid increased the potential and rate of GP. Literatures showed that acid causes hemi-celluloses degradation and NDF reduction leading to GP increase.^39^ Feeds resources with lower NDF have higher potential of GP. Our results are in agreement with Chaji and mohammadabadi, who reported that treating sugarcane pith by sulfuric acid increases GP by rumen bacteria.^40^ Behgar et al. reported that using sulfuric acid + formic acid causes DM degradability increase of alfalfa silage.^41^ Generally, the chemical additives such as acid increase potential and effective degradability of material by rumen microorganisms.^42^

Regardless of the type of the treatment (Table 2), in the present study, the difference between buffalo and cattle rumen bacteria fermentation could be explained by different microbial activity of them. These results are in agreement with those reported by Calabro et al. who found that in vitro GP of forages feeds using inoculums of buffalo rumen is lower than cattle.^43^ Paul and Lal reported that GP by inoculums of buffalo rumen is lower than cattle, which is related to less methane production in buffalo.^44^ However, Chanthakhoun et al. reported that GP by inoculums of buffalo rumen is more than cattle.^45^ As previously mentioned, the reasons of antithetic results can be related to physiological factors such as age, feeding behavior, level of production, animal health, the nature and relationships between different microbial populations and also external factors such as diet composition, nature of feed, feed frequency, dietary changes, change of seasons, changes in day length and geographical factors, which affect the ratio and density of different groups of rumen microorganisms.^13^

Regardless of the type of the animal (Table 2), adding 2.40% sulfuric acid resulted in the highest potential and rate of GP, probably because of degradation of cellulose and lignin ester barriers and subsequent increased biological usability of microorganisms.^46^ Therefore, the results showed that treatment of SCT with 2.40% sulfuric acid or urea + molasses + acid increases GP, may be because of synchronization between readily fermentable carbohydrates of molasses and urea nitrogen and degradation or loosening of lignocellulose bounds.^21^

The PF and microbial‌ biomass efficiency were the highest in buffalo for control, because due to lack of available nutrients (no additive) in control, GP was very low; since PF is calculated as the ratio of truly degraded substrate to GP volume, it also reflects the variation of short chain fatty acids production per unit of degraded substrate.^18^^,^^46^ In cattle, higher PF in treatment containing acid indicated that more organic matter is converted to microbial biomass, so the efficiency of microbial protein synthesis is higher than other treatments. In sulfuric acid treated silages, large amounts of carbohydrates and structural proteins are easily degraded. Therefore, using acid during ensiling, prevents from destroying of proteins, degrades bounds between cellulose, hemicellulose and lignin and subsequently increases the digestibility of DM and crude fiber.^47^ The high part of increasing cell wall degradation is due to degradation of molasses carbo-hydrates, which increases the growth of cellulolytic bacteria. In addition, increased availability of crude protein by adding urea resulted in increased digestibility. Urea in rumen can convert to ammonia, and ammonia is the sole nitrogen source for most cellulolytic bacteria, so urea + molasses increases the amount of cell wall degradation.^48^

Regardless of the type of the treatment (Table 3), microbial biomass and TOMD of cattle were more than buffalo, probably because of higher ability of cattle in cell wall digestibility or more rumen fungi population of cattle.^12^ There is a high negative correlation between structural carbohydrates, particularly lignin, and organic matter (OM) digestibility for both inoculants of cattle and buffalo.^43^ These observations are in agreement with findings of Bhatia et al. who reported that amount of microbial protein synthesis in cattle, based on wheat straw-alfalfa hay-concentrate and wheat straw-clover diets is higher than Indian buffalo.^33^ Kennedy et al. also reported that with fibrous diets, NDF digestibility in buffalo is lower than cattle, probably due to better utilization of cellulose in cattle than buffalo, which is consistent with our results.^31^

The treatments containing urea + molasses + sulfuric acid and sulfuric acid individually (Table 4) showed the highest GP parameters. Treatment with urea caused ammonia production and due to improving of carbo-hydrate and nitrogen synchronization, the accessibility of rumen or silage microbes to cell wall polysaccharides increased.^49^ In the rumen, hydrolysis rate of urea is fast, so released ammonia is not utilized efficiently for synthesis of microbial protein. In order to slow down the release of various complexes of urea, using starch and molasses has been recommended.^19^ In the rumen, synchronization between nitrogen (e.g. urea) and carbohydrate sources (e.g. molasses) is important.^50^ In the present study, using sulfuric acid in silage prevented from degradation of protein and carbohydrates to non-protein nitrogen and organic acids, respectively,^47^ thus nutrients for bacteria increased. Salari showed that treatment of palm leaves with urea and molasses increases OM digestibility that is in accordance with the results of the present experiment.^51^

Regardless of the type of the treatment (Table 4), PF, microbial biomass efficiency and microbial biomass by buffalo were more than cattle. The TOMD in cattle was higher than buffalo. Similar to the present experiment conditions, Rafiee^13^ reported that PF, microbial biomass and microbial biomass efficiency of wheat straw by buffalo are significantly higher than cattle, may be due to higher bacterial populations of buffalo than cattle. Reportedly, the number of cellulolytic bacteria in swamp buffalo is higher than cattle when both are fed with diet based on rice straw.^25^ The majority of microbial studies also showed that under the same nutritional conditions, whole bacterial population and cellulolytic, proteolytic, amylolytic and lipolytic bacteria in buffalo are higher than cattle.^52^

In conclusion, treatments or additives used in the presents study were the proper methods for prolonged preservation of SCT in silo. In aspects of comparison between cattle and buffalo, WRM and bacteria of cattle showed higher digestion of treated SCT silage than buffalo. Addition of sulfuric acid not only had no negative effect on microorganisms, particularly bacteria, but also due to presence of sulfur in sulfuric acid, had positive effect on nutrients digestibility and growth of microorganisms. Thus, using sulfuric acid individually or simultaneously with urea + molasses is the effective and safe method to prepare SCT silage.
